# The psychosocial consequences of covid-19 in adolescents with nonsuicidal self-injury

**DOI:** 10.1186/s13034-023-00566-2

**Published:** 2023-03-04

**Authors:** M. Zetterqvist, Å. Landberg, L. S. Jonsson, C. G. Svedin

**Affiliations:** 1 Department of Child and Adolescent Psychiatry, Region Östergötland, Linköping, Sweden; 2 Department of Biomedical and Clinical Sciences, Center for Social and Affective Neuroscience, Linköping University, Linköping, Sweden; 3 Department of Social Sciences, Marie Cederschiöld University, Stockholm, Sweden

**Keywords:** Covid-19, Nonsuicidal self-injury, Adolescents, Depression, Emotion regulation

## Abstract

**Background:**

Young people have been especially affected by the psychosocial consequences of the covid-19 pandemic. Covid-19 has potentially also been more stressful for vulnerable groups with mental health problems.

**Methods:**

In this cross-sectional study, the psychosocial effects of covid-19 in a vulnerable group of adolescents with nonsuicidal self-injury (NSSI) from a sample of 1602 Swedish high-school students were examined. Data were collected in 2020 and 2021. First, adolescents with and without NSSI were compared on how they perceived the psychosocial impact of covid-19, and second, a hierarchical multiple regression analysis was used to examine whether lifetime NSSI experience was associated with perceived psychosocial consequences of covid-19, when demographic variables and symptoms of mental health problems were controlled for. Interaction effects were also examined.

**Results:**

Significantly more individuals with NSSI reported being burdened by covid-19 compared to those without NSSI. When demographic variables and mental health symptoms were controlled for, adding NSSI experience did not, however, increase the amount of variance explained in the model. The total model explained 23.2% of the variance in perceived psychosocial impact of covid-19. Perceiving the family’s financial situation as poor and neither good nor bad, studying a theoretical high school program, symptoms of depression and difficulties with emotion regulation were significantly associated with perceived negative psychosocial impact of the covid-19 pandemic. There was a significant interaction effect between NSSI experience and depressive symptoms. The effect of NSSI experience was larger when depressive symptoms were lower.

**Conclusions:**

Lifetime NSSI experience in itself was not associated with psychosocial covid-19 consequences when other variables were controlled for, whereas symptoms of depression and difficulties with emotion regulation were. Results imply that vulnerable adolescents with mental health symptoms need special attention and access to mental health support in order to prevent further stress and worsening of mental health symptoms in the wake of the covid-19 pandemic.

## Background

The covid-19 pandemic has impacted the lives of people around the world and increased risk factors for mental health problems in an unprecedented way in modern times [1]. Social interactions are pivotal for the well-being of humans and a lack of social interactions can have a profound effect on our lives. Although the full scope of the long-term psychosocial health effects of the pandemic has yet to be seen, research in the aftermath of covid-19 suggests that children and adolescents are particularly affected by the psychosocial consequences of covid-19 [2–4]. Although less burdened by somatic complications caused by the virus, factors such as societal restrictions, closed schools and family distress have contributed to the lives of children and adolescents being severely affected by covid-19 [5, 6]. In adolescents, elevated levels of depression, anxiety and stress, for example, have been found during the pandemic [1, 4, 7], as well as increased risk behaviors [8].

Different adverse psychosocial consequences of covid-19, such as social isolation and home schooling, are likely influenced by pre covid-19 status. In several mental health conditions there is a bidirectional relationship between vulnerability and stress, where existing symptoms of mental health problems influence how the stress situation is perceived and dealt with, and the stressful experience reciprocally increases symptoms and further risk for mental health problems [9]. Recent research does in fact suggest that the psychosocial effects of covid-19 have been more stressful for already vulnerable groups with existing mental health problems [10], which also seems to be the case for adolescents [7]. Increased loneliness and depressive symptoms in the wake of covid-19 have, for example, shown to be especially evident in those experiencing pre-pandemic risk [11]. In particular, social isolation, parental stress, low socioeconomic status, preexisting mental health conditions and disabilities contribute to increased impairment from covid-19 [12]. There are, however, some inconsistencies in the literature so far. Contrary to expectation, Hamza and colleagues, for example, found that students without preexisting mental health problems experienced worse consequences of the pandemic [13]. Still, the knowledge of how different vulnerable groups, such as adolescents with nonsuicidal self-injury (NSSI), experience the psychosocial impact of the covid-19 pandemic is scarce.

NSSI is often triggered by stress, such as social exclusion, and is conceptualized as a coping strategy [14, 15]. In the light of this, concern has been raised as to how covid-19 has impacted NSSI [5, 10, 16]. NSSI is a common behavior in adolescents [17] and prevalence rates in community samples are reported to be around 20% [18], while rates in clinical groups of adolescents are even higher, around 40–80% [19]. Depression and anxiety are common forms of comorbidity [20]. NSSI has its onset at age 12–14, peaks at 15–16 years of age and decreases in late adolescence [17], which implies that adolescence is an especially important time period in which to study NSSI. NSSI is somewhat more common in girls, especially in clinical samples [19]. The fact that NSSI is a risk factor for suicide and is associated with long-term risk [21], makes it a highly relevant behavior to address.

NSSI is associated with emotion dysregulation [22], and most often the behavior is engaged in to relieve negative emotions and thoughts [23, 24]. In addition to regulating internal, emotional states, NSSI is also engaged in to influence the external, social environment [25]. Some examples are: hoping to receive attention, help and understanding, solving an interpersonal conflict, increasing a sense of belonging and reducing social demands, typically when other forms of social communication have not been successful [26]. Individuals with NSSI also perceive themselves as more interpersonally sensitive [27], experience more loneliness [28], have a negative social bias [29] and poorer interpersonal communication and problem-solving skills compared to those without NSSI [14]. According to the vulnerability-stress model [30], vulnerabilities in adolescents with NSSI could potentially make them more susceptible to covid-19 related stress, which in turn negatively affects the symptomatology. It is therefore of interest to illustrate the impact of the psychosocial consequences associated with covid-19 on adolescents with NSSI.

There are some emerging empirical data indicating that adolescents with NSSI are in fact more burdened by the stress of covid-19. In a recent longitudinal study from Italy, adolescents [*N* = 1061, mean age = 15.5 (SD = 0.8), 52.4% females] with earlier NSSI, internalizing symptoms and difficulties with emotion regulation (ER) before the outbreak of covid-19, experienced more covid-19 related stress, which increased the risk of further NSSI [31]. Also, in another longitudinal study from the US, 91 young female adolescents ages 12–16 years were assessed pre and mid pandemic, and those who persisted with NSSI during covid-19 reported more loneliness and stress compared to those who had no experience of NSSI and those who had prior but not ongoing experience of NSSI during the pandemic [32]. The role of ER in relationship to NSSI and covid-19 has also been examined in adolescents [31, 33]. In a Canadian online survey of 809 adolescents (56% females), mean age 15.7 (*SD* = 1.4), Robillard and colleagues, for example, found that difficulties with ER fully mediated the relationship between covid-19 stress and deliberate self-harm, irrespective of intent [33]. From an international perspective, Sweden has had less lock-down and restrictions compared to many other countries, especially where children were concerned, who continued pre-school and school during the pandemic. Swedish high-school students, however, were twice affected by restrictions with closing of schools and distance education during 2020 and 2021 [34].

NSSI often co-exists with other symptoms. In NSSI research it is generally a challenge to disentangle the unique effect of NSSI above and beyond the contribution of other psychosocial symptoms and diagnoses, which therefore also need to be taken into account. Furthermore, NSSI in adolescents is often hidden from others, and far from all cases come to the attention of health care [35]. It is therefore necessary to investigate the effects of covid-19 in community samples.

In this cross-sectional study, we examine how a vulnerable group of adolescents with lifetime experience of NSSI, from a large community-based sample, perceive the psychosocial consequences of covid-19 compared to adolescents without NSSI, taking other symptoms of mental health problems into account, by examining the following research questions:

First, do adolescents with experience of lifetime NSSI differ in the way they perceive the psychosocial impact of the covid-19 pandemic compared to adolescents without NSSI, and second, is NSSI experience associated with perceived psychosocial covid-19 consequences, when demographic variables and symptoms of mental health problems (symptoms of depression and anxiety, and difficulties with emotion regulation) are controlled for?

We will further build on these results and go on to examine potential interaction effects between NSSI and the mental health problems that turn out to be significantly associated with covid-19 related stress in the analysis.

Based on a vulnerability and stress perspective and earlier research that suggest that the psychosocial effects of covid-19 are potentially more stressful for vulnerable groups, we hypothesized that those with NSSI would experience worse consequences of covid-19. In this cross-sectional study, we also hypothesized that NSSI experience, together with symptoms of depression and anxiety and difficulties with emotion regulation (ER), would be associated with perceived psychosocial impact of covid-19 in adolescents.

## Methods

### Procedure

Data were collected online in classrooms and during home studies in a representative sample of third year students in Swedish high schools during 2020 and 2021. The schools were selected based on information from the national school register and stratified to represent a normal population of third year Swedish high school students regarding school size (three strata) and study program (three strata), rendering nine different types of schools where the geographical distribution across Sweden was also considered. Principals of selected schools were informed by mail and contacted by phone. If no contact was established, another school from the same strata, according to the stratification, was contacted. Participation was voluntary, and if the principal agreed, a date was set for gathering information through an online questionnaire that was filled in during lecture time in the presence of field workers. Students received written information and gave their consent by answering the questionnaire.

A total of 210 schools with 7752 students were selected and of these, 110 schools with 3286 students completed the questionnaire. The typical reasons for schools' non-participation were lack of time and that the schools had to prioritize education and avoid the spread of infection, due to the uncertainty surrounding the covid-19 pandemic. Due to covid-19, the data collection was interrupted, extended, and changed to an online version that was possible to access from home. This resulted in three periods of data collection during 2020 and 2021, the spring of 2020 (*n* = 1195), the autumn of 2020 (*n* = 737), and the spring of 2021 (*n* = 1350). Four non-serious questionnaires were excluded rendering 3282 students and yielding a response rate of 42.3%. The first data collection from the spring of 2020 did not include questions on the impact of the covid-19 pandemic and were excluded from further analysis, resulting in 2087 participants. Three extreme outliers on the covid-19 items were excluded after manual examination. Twenty participants had not answered the NSSI question, making group categorization impossible and they were therefore excluded, resulting in 2064 individuals. Furthermore, after participants that had chosen the “I don’t know” option (*n* = 443 for covid-19 items and *n* = 19 for perception of family’s financial situation) were excluded, a final sample of 1602 participants remained for the analyses.

The study was approved by the Swedish Ethical Review Authority (2019-05013-31, 2020-03611, 2020-06556). Written informed consent from the participants’ legal guardian was not required since all participants were older than 15 years, in accordance with the national legislation. During data collection, participants were offered written information on where they could seek professional help if needed.

### Participants

In the present study, 1602 adolescent high-school students (*M* = 18.1, *SD* = 0.6) were included. Of these, 681 (42.5%) identified as boys, 903 (56.4%) as girls and 18 (1.1%) as non-binary. Lifetime prevalence of NSSI was reported by 488 (30.5%) and 1114 (69.5%) reported no experience of NSSI. For participants’ demographic data, see Table 1.

**Table 1:** Demographic information in adolescents with and without NSSI (N = 1602)

	Total sample *N* = 1602 *n* (%)	NSSI *n* = 488 *n* (%)	No NSSI *n* = 1114 *n* (%)	Stats NSSI vs. no NSSI
Gender				
Boy	681 (42.5)	121 (24.8)	560 (50.3)	*χ*^2^ [2] = 102.58, *p* < .001, Cramer’s V = .25
Girl	903 (56.4)	353 (72.3)	550 (49.4)	
Non-binary identification	18 (1.1)	14 (2.9)	4 (0.4)	
Age (*m*, *sd*)	18.1 (0.6)	18.1 (0.6)	18.1 (0.6)	ns
Study program				
Theoretical	1127 (70.3)	308 (63.1)	819 (73.5)	*χ*^2^ [2] = 18.30, *p* < .001, Cramer’s V = .11
Practical	446 (27.8)	171 (35.0)	275 (24.7)	
Lacks formal merits for high-school	29 (1.8)	9 (1.8)	20 (1.8)	
Fathers working	1396 (87.1)	416 (85.2)	980 (88.0)	ns
Mothers working	1410 (88.0)	420 (86.1)	990 (88.9)	ns
Fathers with university education	643 (40.1)	173 (35.5)	470 (42.2)	ns
Mothers with university education	903 (56.4)	268 (54.9)	635 (57.0)	ns
Financial situation in the family				
Good	1220 (76.2)	316 (64.6)	904 (81.1)	*χ*^2^ [2] = 51.38, *p* < .001, Cramer’s V = .18
Neither good nor bad	321 (20.0)	141 (28.9)	180 (16.2)	
Poor	61 (3.8)	31 (6.4)	30 (2.7)	
Adolescents born in Sweden	1459 (91.0)	443 (90.8)	1015 (91.1)	ns
Fathers born in Sweden	1326 (82.8)	411 (84.2)	915 (82.1)	ns
Mothers born in Sweden	1302 (81.3)	404 (82.8)	898 (80.6)	ns
Living situation				
With both parents	997 (62.2)	261 (53.5)	736 (66.1)	*χ*^2^ [4] = 38.65, *p* < .001, Cramer’s V = .16
Alternating between both parents	190 (11.9)	64 (13.1)	126 (11.3)	
With one parent with or without new partner	327 (20.4)	118 (24.2)	209 (18.8)	
Alone or with siblings or partner	77 (4.8)	43 (8.8)	34 (3.1)	
In foster care or institution	11 (0.7)	2 (0.4)	9 (0.8)	

NSSI = nonsuicidal self-injury

### Measures

This study emanates from the survey “Young people, sex and the internet after #metoo” [36] and the questionnaire comprised 110 main questions concerning sociodemographic background, experiences of abuse, risk behaviors, psychosocial health, impact of covid-19 and #metoo. In the present study, questions relating to sociodemographic background, NSSI, impact of covid-19, and symptoms of depression and anxiety, as well as ER were used.

#### Demographic information

Demographic questions were created for the purpose of the study assessing characteristics such as gender, age, type of education, parents’ occupation and education, perception of family’s economy, own and parents’ immigrant background and living situation. Adolescents self-reported demographic information in fixed answer categories (Table 1).

#### Nonsuicidal self-injury

Lifetime prevalence of NSSI (yes/no) was assessed with the NSSI item from the Self-Injurious Thoughts and Behaviors Interview (SITBI; [37]), short-form and self-report version: *“Have you ever actually engaged in non-suicidal self-injury (NSSI; that is, purposely hurt yourself without wanting to die, for example by cutting or burning)?”.*

#### Covid-19 impact

Questions on the impact of covid-19 psychosocial stress were created for the purpose of the study, assessing perceived overall effect of the covid-19 pandemic on loneliness, home education, relationships with family and peers, and health care support/treatment (See Table 2). Answers were assessed with fixed answer categories on a Likert scale ranging from 1 to 5 (totally disagree to totally agree). The seven items included in this study to assess psychosocial consequences of covid- 19 ranged from 7 to 35, with higher scores indicating a higher perceived psychosocial impact of covid-19. Cronbach’s alpha was 0.77, indicating acceptable internal consistency for the seven items. In an exploratory factor analysis (principal axis) with varimax rotation, all seven items loaded on one underlying factor (eigenvalue 3.0) that explained 43.4% of the variance with factor loadings for the seven items ranging between 0.34 and 0.80. In the questionnaire, there was an “I don’t know” option, which was excluded from analyses.

**Table 2:** Self-reported psychosocial impact of covid-19 in adolescents with and without NSSI (*N* = 1602)

I agree/totally agree that the covid-19 pandemic has…	NSSI *n* = 488 *n* (%)	No NSSI *n* = 1114 *n* (%)	Stats NSSI vs. no NSSI
…affected me a lot	365 (74.8)	759 (68.1)	*χ*^2^ [2] = 7.93, *p* = .02, Cramer’s V = .07
I agree/totally agree that the covid-19 pandemic has affected me so that:			
I have felt more alone than before	271 (55.5)	429 (38.5)	*χ*^2^ [2] = 44.10, *p* < .001, Cramer’s V = .17
It has become more difficult to discuss with friends if anything worries me	157 (32.2)	200 (18.0)	*χ*^2^ [2] = 43.29, *p* < .001, Cramer’s V = .16
It has become more difficult to contact adults outside the family if anything worries me	156 (32.0)	189 (17.0)	*χ*^2^ [2] = 57.78, *p* < .001, Cramer’s V = .19
My situation at home has become worse than before	102 (20.9)	79 (7.1)	*χ*^2^ [2] = 83.00, *p* < .001, Cramer’s V = .23
Support and treatment have been interrupted or postponed	117 (24.0)	98 (8.8)	*χ*^2^ [2] = 79.02, *p* < .001, Cramer’s V = .22
Home education has not worked out well for me	207 (42.2)	342 (30.7)	*χ*^2^ [2] = 20.74, *p* < .001, Cramer’s V = .11

NSSI = nonsuicidal self-injury. Crosstab analysis with chi square based on three categories: agree/totally agree, disagree/totally disagree and in between

#### Symptoms of depression and anxiety

Symptoms of depression and anxiety were measured using the depression and anxiety subscales from the Trauma Symptom Checklist for Children (TSCC; [38]). The depression and anxiety subscales include nine items each. Response options are “never”, “sometimes”, “often” and “almost all of the time” and scores range from 0 to 27 for each subscale. Higher scores indicate higher levels of depressive symptoms and anxiety symptoms. The Swedish translation by Nilsson et al. [39] was used. Cronbach’s alpha in the present sample was 0.90 for the subscale depression, and 0.86 for anxiety, indicating good to excellent internal consistency for the two subscales.

#### Difficulties with emotion regulation scale

Difficulties with emotion regulation were measured using the 16-item version (DERS-16; [40]). DERS-16 is a brief version of the 36-item original DERS version [41] and consists of 16 items rated on a five-point Likert scale from “almost never” to “almost always.” Scores range from 16 to 80, where higher scores indicate more difficulties with regulating emotions. Cronbach’s alpha in the present sample was 0.95 for the total scale, indicating excellent internal consistency.

### Data analysis

Data were analyzed with descriptive statistics using frequencies, percentages, mean values and standard deviations, and cross-tabulation with chi-square for categorical data and independent samples t-test for group comparisons of continuous data. There were no missing data in the analyses. Cramer’s V, and Cohen’s were used for effect sizes with 0.07, 0.21 and 0.35 and 0.2, 0.5 and 0.8, indicating small, medium and large effect [42] for Cramer’s V and Cohen’s, respectively. Hierarchical multiple regression was used with psychosocial impact of covid-19 as continuous dependent variable (DV) and demographic variables entered at step 1; symptoms of depression, symptoms of anxiety and difficulties with ER entered at step 2; and, finally, NSSI experience was entered at step 3, as independent variables (IV). Moderation (model 1) was tested using the bootstrapping method and the 95% CI with 5000 bootstrapping resamples. All statistical analyses were performed using the SPSS 28.0 software package (SPSS Inc, Chicago, IL) with process macro 4.0 downloaded from Hayes [43].

## Results

### Perceived psychosocial impact of covid-19 in adolescents with and without NSSI

Responses on covid-19 items were categorized into “agree/totally agree”, “disagree/totally disagree” and “in between” and presented based on group classification (NSSI experience or no NSSI experience). See Table 2. Of those with NSSI, 74.8% agreed/totally agreed that the covid-19 pandemic had affected them a lot, compared to 68.1% in the group without NSSI. More participants with NSSI also agreed/totally agreed that they felt more alone during the pandemic than they had done before (55.5 vs 38.5%), that it had become more difficult to discuss with friends if anything worried them (32.2% vs 18.0%), and also that contacting adults outside the home if anything worried them had become more difficult (32.0% vs 17.0%), compared to those without NSSI. Significantly more participants with NSSI (20.9%) agreed that the situation at home had become worse during the pandemic, compared to those without NSSI (7.1%). Of those with NSSI, 24.0% agreed or totally agreed that support and treatment had been interrupted, and the equivalent number for those without NSSI was 8.8%. Concerning participants’ perception of home education, more adolescents with NSSI (42.4%) reported that this had worked out badly or very badly for them (compared to 30.7% of those with no NSSI). Cross-tabulation with chi-square analyses showed that all group differences in experience of psychosocial impact of covid-19 were statistically significant (ranging from *p* = 0.02 to *p* < 0.001). See Table 2. ES for “situation at home having become worse” and “support and treatment having been postponed” were medium sized (Cramer’s V = 0.23 and 0.22, respectively). For all other comparisons the ES were small, ranging from Cramer’s V = 0.07 to 0.19. Applying Bonferroni correction for multiple comparisons resulted in an alpha value of *p* = 0.007, which meant that group differences on the item “the pandemic has affected me a lot” (*p* = 0.02), no longer reached statistical significance.

Using the total covid-19 impact as a continuous variable, those with NSSI reported a mean average of *M* = 20.2 (*SD* = 6.4) and those without reported *M* = 16.6 (*SD* = 5.6), which was a statistically significant difference (*p* < 0.001), with a Cohen’s ES of 0.62 (medium effect). The mean average of reported symptoms of depression in those with NSSI was significantly (*p* < 0.001) higher (*M* = 11.2, *SD* = 5.7 vs. *M* = 5.2, *SD* = 4.2) compared to those without NSSI with a Cohen’s ES of 1.28 (large effect). Symptoms of anxiety was also significantly higher in those with NSSI (*M* = 9.1, *SD* = 5.3 vs. *M* = 4.8, *SD* = 4.0, *p* < 0.001, Cohen’s ES = 0.96) with a large ES, as was difficulties with ER (*M* = 47.9, *SD* = 15.5 vs. *M* = 32.1, *SD* = 13.2, *p* < 0.001, Cohen’s ES = 1.13). See Table 3.

**Table 3:** Self-reported psychosocial impact of covid-19 and symptoms in adolescents with and without NSSI (*N* = 1602)

	NSSI *n* = 488 *m* (*sd*)	No NSSI *n* = 1114 *m* (*sd*)	Stats NSSI vs. no NSSI
Impact of covid-19	20.2 (6.4)	16.6 (5.6)	*t*(1600) = 11.42, *p* < .001, Cohen’s d = .62
Symptoms of depression	11.2 (5.7)	5.2 (4.2)	*t*(1600) = 23.58, *p* < .001, Cohen’s d = 1.28
Symptoms of anxiety	9.1 (5.3)	4.8 (4.0)	*t*(1600) = 17.71, *p* < .001, Cohen’s d = .96
Difficulties with emotion regulation	47.9 (15.5)	32.1 (13.2)	*t*(1600) = 20.87, *p* < .001, Cohen’s d = 1.13

NSSI = nonsuicidal self-injury. Higher scores indicate more difficulties/symptoms

### Association between NSSI and perceived covid-19 impact controlling for demographics and mental health

A hierarchical linear regression was used to assess the association between lifetime NSSI experience and perceived psychosocial impact of covid-19, after demographic variables and symptoms of mental health had been controlled for. Preliminary analyses were conducted to ensure that the assumptions of normality, linearity, multicollinearity and homoscedasticity were not violated. The demographic variables that were significantly different between adolescents with and without experience of NSSI (see Table 1): gender, study program, family financial situation and living situation were entered at step 1. Dummy variables were created for these categorical variables. The demographic variables together explained 8.1% of the variance in perceived covid-19 impact and the model was significant (*R* squared change = 0.08, *F* change [8, 1593] = 17.45, *p* < 0.001). See Table 4. After entry of symptoms of depression and anxiety, and difficulties with ER at step 2, the total variance explained by the model was 23.1%. These symptoms thus explained an additional 15.1% of the variance in covid-19 impact, after controlling for demographic variables, *R* squared change = 0.15, *F* change [3, 1590] = 104.00, *p* < 0.001. At the third and last step, NSSI experience was entered. Adding NSSI did not increase the variance explained in impact of covid-19 pandemic (23.2%), after controlling for demographics and symptoms of mental health (*R* squared change = 0.00, *F* change [1, 1589] = 2.08, *p* = 0.15). The variance explained by the total model was 23.2%, *F*[12, 1589] = 40.09, *p* < 0.001. In the final model, experiencing the family’s financial situation as poor and neither good nor bad, studying a theoretical high school program, difficulties with ER and symptoms of depression were statistically significant (Table 4).

**Table 4:** Hierarchical multiple regression for perceived negative psychosocial consequences of covid-19

	Perceived negative consequences of covid-19						
Block of predictors	*R*^*2*^	*R*^*2*^ *change*	*B*	*SE B*	*β*	*t*	*p*
Demographic background variables	0.08	0.08***					
Gender (girl)*			0.52	0.31	0.04	1.69	0.09
Gender (non-binary)*			1.09	1.31	0.02	0.83	0.41
Study program (theoretical)˜			0.87	0.31	0.07	2.84	**0.005**
Study program (individual⁑)˜			0.67	1.03	0.02	0.65	0.52
Family financial situation (poor)'			1.64	0.73	0.05	2.24	**0.03**
Family financial situation (neither good nor bad)'			0.87	0.36	0.06	2.42	**0.02**
Living conditions (one parent)†			−0.29	0.35	−0.02	−0.83	0.41
Living conditions (other‡)†			−0.26	0.60	−0.01	−0.43	0.67
Self-reported symptoms	0.23	0.15***					
Depression			0.26	0.05	0.24	5.86	** < 0.001**
Anxiety			0.07	0.05	0.06	1.55	0.12
Difficulties with emotion regulation			0.07	0.01	0.17	5.23	** < 0.001**
Nonsuicidal self-injury	0.23	0.00	0.50	0.35	0.04	1.44	0.15

Boldface values represent unique relationship between predictor and perceived consequences of covid-19, all statistics (except *R*^2^ and *R*^2^ change) presented in the table refer to the final third model

^***^ *p* < 0.001

^*^reference category for dummy coding is boy

˜reference category for dummy coding is practical study program

'reference category for dummy coding is good financial situation

†reference category for dummy coding is living with both parents together or alternating

⁑individual study program lacks formal merits for high-school

‡other living conditions refers to living alone, with siblings or partner, or in foster care/institutional care

### Follow-up analyses of interaction effects

**Fig. 1:**
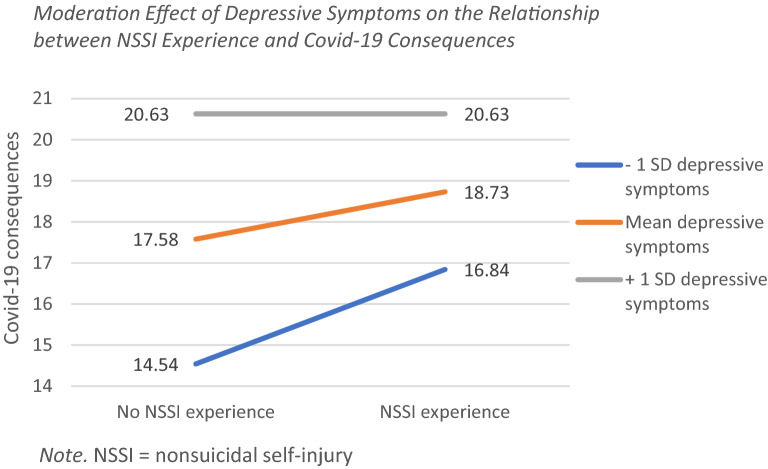
Moderation Effect of Depressive Symptoms on the Relationship between NSSI Experience and Covid-19 Consequences

Since depressive symptoms and difficulties with ER were significant predictors for covid-19 stress, we went on to exploratively test interaction effects with NSSI. First, to examine whether the relationship between NSSI experience and perceived psychosocial impact of covid-19 was influenced by depressive symptoms, a moderator analysis was performed with covid-19 consequences as DV, NSSI experience as IV and depressive symptoms as moderator. Depressive symptoms were significant (B = 0.56 [0.48, 0.63], *p* < 0.001), as was NSSI experience (B = 2.62 [1.44, 3.79], *p* < 0.001). The interaction between depressive symptoms and NSSI experience was also significant (B = −0.21 [−0.32, −0.10], *p* < 0.001). Depressive symptoms dampened the effect of NSSI experience on covid-19 consequences. The conditional effects showed similar results. The effect of NSSI experience on covid-19 consequences was highest (2.30 [1.26, 3.34], *p* < 0.001) with lower depressive symptoms (−1 SD: 1.52), decreased (1.15 [0.46, 1.84], *p* = 0.001) with mean levels of depressive symptoms (6.99) and was no longer significant (−0.00, [−0.81, 0.80], *p* = 0.99) with higher levels of depressive symptoms (+ 1 SD: 12.47). See Fig. 1 for slopes.

**Fig. 2:**
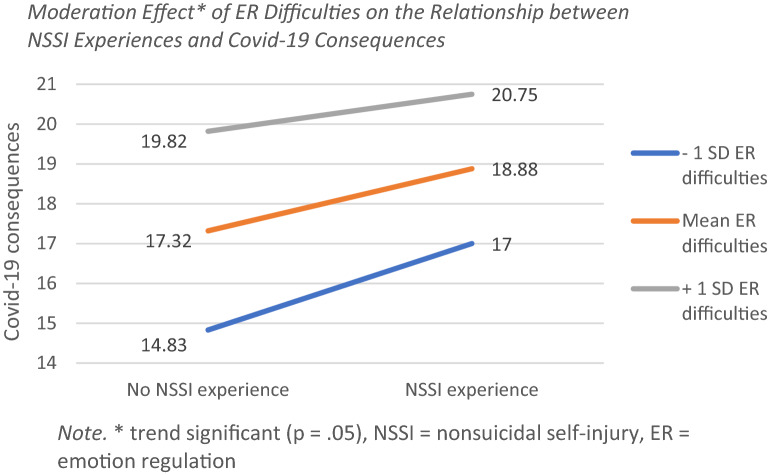
Moderation Effect* of ER Difficulties on the Relationship between NSSI Experiences and Covid-19 Consequences

Second, the interaction effect between ER difficulties and NSSI experience on covid-19 stress was examined. In the moderator analysis with covid-19 consequences as DV and NSSI experience as IV and difficulties with ER as moderator, ER difficulties were significant (B = 0.16 [0.13, 0.18], *p* < 0.001), as was NSSI experience (B = 3.00 [1.20, 4.80], *p* = 0.001). The interaction between difficulties with ER and NSSI experience was not significant (B = −0.04 [−0.08, 0.00], *p* = 0.05), but showed a trend. Since there was a trend, conditional effects are presented. The effect of NSSI was highest (2.17 [1.11, 3.23] *p* < 0.001) with lower levels of ER difficulties (−1 SD: 21.23), decreased (1.55 [0.86, 2.24] *p* < 0.001) with mean levels of ER difficulties (36.96) and decreased further (0.93 [0.15, 1.72] *p* = 0.02) with higher levels of ER difficulties (+ 1 SD: 52.68). See Fig. 2 for slopes.

## Discussion

In this cross-sectional study of a large community sample of older adolescents (*N* = 1602), negative psychosocial impact of the covid-19 pandemic was examined in individuals with and without NSSI. Adverse effects of covid-19 were more pronounced among adolescents with NSSI. Those with NSSI experienced more covid-19 related stress, such as loneliness, and less ability to reach out to friends and adults outside of the home if needed. Furthermore, more adolescents with NSSI had experienced an interruption in treatment and that home schooling had not worked well. Those with NSSI experience also reported higher levels of anxiety, depressive symptoms and ER difficulties compared to those without NSSI. When demographic variables and symptoms of depression, anxiety and emotion dysregulation were controlled for in a hierarchical regression, NSSI experience was not, however, independently associated with psychosocial covid-19 consequences. In the final model, experiencing the family’s financial situation as poor and neither good nor bad, studying a theoretical high school program, difficulties with ER and symptoms of depression were statistically significant. Furthermore, there was an interaction effect between NSSI and depressive symptoms, and a trend (*p* = 0.05) for an interaction effect between NSSI experience and ER difficulties on covid-19 consequences, where higher levels of depressive symptoms and ER difficulties weakened the effect of NSSI experience.

First, higher levels of covid-19 stress were reported by individuals with NSSI. That individuals with NSSI reported more adverse effects of covid-19 than their non-self-injuring peers confirms our hypothesis and also earlier studies that have shown that covid-19 stress has a more adverse effect on already vulnerable populations [7, 11]. Increased loneliness, for example, in association with covid-19 has previously been found to be especially evident in those experiencing pre-pandemic risk [11]. In the current study, more adolescents with NSSI also reported loneliness during the covid-19 pandemic. Furthermore, adolescents with NSSI reported that the pandemic had made it more difficult for them to connect with peers and adults outside the family if anything worried them. This is especially concerning since social support has shown to have a beneficial effect on NSSI, whereas perceived low social support, especially in the face of adversities, is associated with an increased risk for NSSI [44]. That treatment had been interrupted is also disconcerting and potentially implies that transition to online resources, for instance, has not been unproblematic for this vulnerable group. The need for digital options for support and treatment of NSSI in the context of the pandemic has previously been highlighted [45].

Adolescents with experience of NSSI thus perceived more covid-19 related stress compared to those without NSSI, as well as reporting higher levels of depressive and anxiety symptoms and difficulties with ER, which is consistent with previous research [20, 22, 46]. Adolescents without vulnerabilities and mental health problems are potentially better equipped with alternative coping strategies, and thus less susceptible to the negative consequences of the stressor, according to the vulnerability-stress model.

Another potentially negative consequence for adolescents with NSSI related to covid-19 could be that prescribed restrictions resulted in limited access to necessary health-promoting activities. Different forms of behavioral activation, engaging in social activities, meeting friends and pursuing hobbies and interests, for example, are activities that can be used to help prevent an episode of NSSI, and also contribute to a positive identity and sense of empowerment [47]. Limited opportunity to engage in these primarily social activities could potentially lead to a vicious cycle, with increased risk for further mental health problems in vulnerable groups.

Second, we examined if NSSI independently was associated with covid-19 related stress, even when other variables were controlled for. There is recent empirical support that adolescent girls might be more negatively impacted by the covid-19 pandemic, with a more risky life style [8], more stress [1] and increased presentation to health care for self-harm, irrespective of intent [48], but our results did not support any sex differences in how covid-19 was perceived when symptoms of depression, anxiety and ER difficulties were taken into account.

The consequences of covid-19 are unfairly distributed in the population [49], and in this study, perceiving the family financial situation as poor and neither good nor bad were associated with negative psychosocial consequences of the covid-19 pandemic. That the effects of the pandemic particularly affected socioeconomically burdened groups has been shown earlier in children [50]. Studying a theoretical high school program was also associated with higher levels of covid-19 related stress. Few other studies have investigated this, but one tentative speculation could be that the pandemic caused more stress for adolescents studying theoretical programs, due to its potentially negative impact on their chance to applying to university.

When symptoms of depression, anxiety and difficulties with ER were controlled for, NSSI experience in itself, entered last in the model, was not an independent significant predictor in the total model on covid-19 consequences, and our hypothesis was thus not confirmed. Symptoms of depression and difficulties with ER explained more of the variance in covid-19 psychosocial stress than NSSI experience, and thus seem more important than NSSI itself when it comes to covid-19 consequences. Symptoms of depression and difficulties with ER were thus significant predictors in the multiple regression model, which was in line with our hypothesis. Symptoms of anxiety, however, were not associated with covid-19 stress, contrary to our hypothesis. It has previously been shown that adolescents with depressive symptoms [7] have been more affected by the pandemic, which our results confirm. ER difficulties have also earlier been found to be important in relation to covid-19 stress [31, 33]. The total explained variance in the model (23%) in the current study was low, indicating that other variables than those entered into the model also contribute to the understanding of the psychosocial impact of covid-19. Thus, how the covid-19 stressor is perceived does not seem to be limited to NSSI experience in itself, but rather to other symptoms, such as symptoms of depression and difficulties with ER. An earlier longitudinal study of Italian adolescents showed that earlier NSSI, together with internalizing symptoms and difficulties with ER, predicted more covid-19 related stress [31].

Since NSSI in itself was not sufficient to predict perceived covid-19 related stress when other variables were controlled for in our model, and symptoms of depression and difficulties with ER were significant, we went on to further examine the relationship between NSSI, symptoms and covid-19 related stress by testing interaction effects. Results showed that depressive symptoms weakened the effect of NSSI experience. The impact of NSSI experience on covid-19 stress depended on whether levels of depressive symptoms were higher or lower. The effect of lifetime NSSI experience on covid-19 stress was higher when depressive symptoms were lower. When depressive symptoms were higher, on the other hand, the importance of NSSI experience decreased. NSSI experience thus interacted differently with covid-19 stress depending on the level of depressive symptoms. Higher symptoms of depression thus played a more important role in relationship to covid-19 related stress, which suggests that higher depressive symptoms, which are more pervasive in nature than lifetime prevalence of NSSI behavior, implies a greater burden, while the effect of NSSI was higher when depressive symptoms were lower. There was, however, no significant interaction effect between NSSI experience and ER difficulties. There was, however, a trend (*p* = 0.05), where ER difficulties weakened the effect of NSSI experience. When levels of ER difficulties were higher, the effect of NSSI was weakened. Since the interaction was only trend significant, caution has to be taken when interpreting these results. In conclusion, NSSI and depressive symptoms interacted to explain how covid-19 stress was perceived, i.e., with higher depressive symptoms, the effect of NSSI disappeared.

By international comparison, Sweden has had less lock-down and restrictions and there might be cultural differences in how much the pandemic affects adolescents [51], which limits comparisons between countries. The lives of high-school students in Sweden have, however, been affected with distance education. Despite including a large sample of Swedish high school adolescents that enabled sub-group analysis, the study is not without limitations. The main limitation is the cross-sectional design, which does not allow for causal inferences. There is potentially a bidirectional relationship between vulnerability, symptoms and experience of covid-19 stress, but the design cannot cater for such complexity. The precise nature of these relationships is therefore unclear. Another limitation in the current study is related to the aspect of time. The data only allow for analysis of lifetime prevalence of NSSI, while ER difficulties and symptoms of depression and anxiety were rated currently, and this imbalance could potentially influence the results. The single item measurement of lifetime NSSI, which does not allow for analyses of NSSI frequency, severity or whether NSSI was ongoing or in the past, is another limitation. The fact that the study was conducted in the midst of the pandemic is a strength, contributing important information about this unique time period. However, at the same time it also means that measures of covid-19 impact had not yet been well validated. The measure of psychosocial consequences of covid-19 was developed for the purpose of the study, which calls for some caution. However, a factor analysis showed that all seven items loaded on one underlying factor. Furthermore, the response rate was low, which limits generalization to all high school students. Lack of time and covid-19 were most often given as reasons for schools not participating in the data collection. However, the data do not allow for further analysis of potential systematic bias in school drop-out. Similarly, data on the adolescents who did not choose to take part in the study are also lacking, although those reluctant to take part generally tend to be a more burdened group, which most likely would increase group differences had they been included. The results cannot be generalized beyond community samples of older adolescents. However, schools were selected based on information from the national school register and stratified to represent a normal population of third year Swedish high school students regarding school size and study program where the geographical distribution across Sweden was also considered.

## Conclusions

Taken together, adolescents with experience of NSSI perceived more negative psychosocial consequences of covid-19 than adolescents without NSSI experience. In this cross-sectional study, NSSI experience in itself did not, however, independently predict psychosocial covid-19 consequences when other variables were controlled for, whereas other variables, such as symptoms of depression and difficulties with emotion regulation did. There was a significant interaction effect between NSSI experience and depressive symptoms, where the effect of NSSI experience depended on the severity of the depressive symptoms. Results from this study thus preliminary suggest that vulnerable groups are especially susceptible to the psychosocial consequences of covid-19, and therefore need special attention and access to mental health support in order to prevent further stress and deterioration of mental health in the wake of the covid-19 pandemic [52]. Examples of such interventions could be to routinely screen for how vulnerable adolescents with symptoms have perceived the psychosocial consequences of covid-19 and work to increase social support for these groups, as well as focusing on skills for dealing with social stress to minimize the impact of the covid-19 pandemic and prevent further risk of mental health problems.

## Data Availability

Data is not available since we do not have participants’ or ethical permission to share data.
